# Implementing Buurtzorg-derived models in the home care setting: a Scoping Review

**DOI:** 10.1016/j.ijnsa.2022.100061

**Published:** 2022-01-12

**Authors:** Anna Hegedüs, Anita Schürch, Iren Bischofberger

**Affiliations:** a Research Institute, Careum School of Health, part of Kalaidos University of Applied Sciences, Zurich, Switzerland; b Bern University of Applied Sciences, Departement of Health Professions, Applied Research & Development in Nursing, Bern, Switzerland; c Bern University of Applied Sciences, Departement of Health Professions, Competence Centre Participatory Health Care, Bern, Switzerland

**Keywords:** Buurtzorg, Home care, Community care, Nursing, Self-management, Implementation, Experience, Scoping Review

## Abstract

**Background:**

Buurtzorg is a pioneering healthcare organization founded in the Netherlands. Buurtzorg has established independent, self-managing teams of nurses and promises high-quality home care at a lower cost through person-centered care, continuity of care, building trusting relationships, and networks in the neighborhood. Traditional home care services are increasingly reorganized according to the Buurtzorg-principles.

**Objectives:**

This review aims to describe the experiences gathered during the implementation of the Buurtzorg-derived model outside the Netherlands. It further outlines their potential effects.

**Design:**

Scoping Review

**Methods:**

Several electronic databases were searched for relevant articles, supplemented by hand-searching and internet searches for gray literature. Various publication types that described the implementation of the Buurtzorg model or its outcomes in countries other than the Netherlands were included. The data were analyzed using qualitative content analysis. Two authors coded the data in several cycles and created categories and subcategories.

**Results:**

The review identified 25 publications reporting the experiences or outcomes of Buurtzorg-derived models outside the Netherlands. Upon implementing elements of Buurtzorg, the home care organizations adopted a person-centered care approach, with improved communication with patients and family caregivers, and could establish new networks with other services. The main challenges were related to the self-managed working culture, the organizational framework, or national healthcare policies, which hindered the implementation process.

**Conclusions:**

The implementation of Buurtzorg-derived models is complex, challenging, and requires adaptations on several levels: upskilling of networking and staffing competencies in teams, leadership and IT requirements in the organization, and policy changes in the healthcare system. Individualized approaches and solid conceptual preparation are required for implementation.

**Tweetable abstract:**

Experiences with the implementation of Buurtzorg in home care services outside the Netherlands. A scoping review @HegeduesAnna

What is already known

• The Dutch home care organization, Buurtzorg, attracted international attention for delivering high-quality care at a lower cost than most competing organizations.
• Early efforts are underway to adapt the Buurtzorg approach to local circumstances in several countries.

What this paper adds

• A wide range of scientific and non-scientific publications have reported on the experiences of home care services implementing Buurtzorg-derived models outside the Netherlands. Studies focusing on their effects are scarce.
• The main challenges in the implementation process are related to the self-managed working culture, organizational frameworks, and national healthcare policies that hinder the implementation process.

## Background

One of the most crucial present and future healthcare challenges is to meet the population's desire for comprehensive home care, including services, until the end of life (Ilinca et al., 2018). Instead of solely focusing on medical diagnoses and therapies, nursing science has always been a strong advocate of comprehensive care, exemplified by Roper et al.'s (1980) model of activities of daily living. However, in the health policy arena of Western countries, decisions curtail these efforts, resulting in unmet needs at home for patients and family caregivers. The COVID-19 crisis has intensified this challenge and highlighted the urgent need for alternatives to hospital care (Jones and Bowles, 2020). Patients during and after the pandemic require individualized and integrated services at all ages and across ill-health conditions, supported by competencies from both health and social care professionals.

Historically, home and community care settings have been organized in small home care services that hire nursing and auxiliary staff. The services are embedded in a geographical area where informal collaboration with local pharmacies and general practitioners, most of them in single-doctor offices as the standard care model, is possible (Tarricone and Tsouros, 2008). In the past few decades, under increasingly specialized, fragmented, and regulated conditions and high financial and staffing pressure, home care services have faced substantial scrutiny. However, person-centered home care that meets patients' and family caregivers' needs, preferences, and values remains the ultimate goal for meeting the population's long-term health care needs (National Institute for Health and Care Excellence, 2015).

Blurred boundaries surrounding where home care takes place were discussed two and a half decades ago, and baseline concepts were analyzed (Kane, 1995): Is home care provided in private households, in-home residential services, or other long-term care facilities? How do regulatory regimes work with regard to quality and reimbursement requirements? The answer to these questions was expanding the concept of home care by rethinking structures and flexibility for services that support self-determination. Over the past two decades, a sharp increase in medical and assistive technology and in information and communication management systems have allowed for a multitude of acute care interventions at the patients' place of living. For example, new technologies include home ventilation (Ergan et al., 2019) and ICT tools used to improve symptoms or movement control at home (Lindberg et al., 2013).

Across healthcare sectors, innovative home care models have constantly been fostered in Europe (van Eenoo et al., 2018) and beyond (Tarricone and Tsouros, 2008). They aim to provide guidance for leadership and practice as well as equal access for all patients in need. Some landmark concepts have been developed that foster good practices in home care. Some are from a hospital perspective, such as Planetree, which supports smooth transitions from the hospital to the private household by establishing systematic patient engagement opportunities preparing for discharge (e.g., Frampton et al., 2017). In long-term care, the concept of feeling well and self-determined at home was created and evaluated in the Green House program with its small-house nursing homes (Kane et al., 2007). It follows the person-centered approach as a shift away from the medical field toward a behavioral model, particularly highlighted in dementia care (Kitwood, 1997). Consistent with these landmark concepts, the Netherland-based Buurtzorg home care model was created as an innovation with regard to staffing and IT support for self-organized teams (Monsen and de Blok, 2013a). It has been discussed as an innovation toward the (re)organization of traditional home care services. This review focuses on this model because it has been well perceived in the home care settings recently in several European countries and beyond. However, its staffing and organizational structure and the option to transfer and implement it in other national healthcare services require in-depth discussions.

## The Buurtzorg model

Buurtzorg is a pioneering health care organization founded in the Netherlands in 2006 (Ćirković, 2018; Monsen and de Blok, 2013b). Since the launch of the initial neighborhood care team ('buurt' = neighborhood, 'zorg' = care), Buurtzorg Netherlands has grown rapidly to over 10,000 nurses and nurse assistants across 850 self-managing neighborhood teams (Buurtzorg, 2021b).

A central tenet of the Buurtzorg model is "humanity over bureaucracy", which is enacted in two key strategies. The first strategy is a holistic and person-centered nursing practice ethos founded on advocating patients' autonomy and empowerment. Core principles are continuity of care, building trusting relationships, building networks in the neighborhood, and linking patients to community resources. The second strategy is to establish independent, self-managing teams of up to 12 nurses, of whom 70% are registered nurses and 40% have a bachelor's degree (Drennan, Ross et al., 2018). The teams are supported by regional coaches and a small number of back-office specialists who centrally deal with finances and administration (Monsen and de Blok, 2013b).

Buurtzorg adopted a flat per-hour payment method for its services. Nurses are entitled to decide the amount of care needed for each patient. An innovative and comprehensive IT system promises transparency and enhanced communication. It allows administrative data to be captured by the back-office team without further recourse to the district nurses (Cavedon et al., 2020; Hamm and Glyn-Jones, 2019). In terms of the cost-effectiveness and satisfaction of patients and caregivers, Buurtzorg achieved positive results (KPMG Plexus, 2015; Monsen and de Blok, 2013b).

However, Buurtzorg was also criticized for selecting complex patients to maximize their revenue and to have difficulty in managing unplanned care. Therefore, patients had to seek help from other home care services (Gray et al., 2015). However, substantial evidence supporting these claims is missing (Gray et al., 2015). Consequently, the business model has gained international attention and recognition for innovating home care (Gray et al., 2015; Jantunen et al., 2020; Leask and Gilmartin, 2019). Over the last few years, a growing number of initiatives have sought to adapt and adopt the Buurtzorg model in various countries (Kreitzer et al., 2015; Sheldon, 2017). Further research is needed to investigate Buurtzorg-derived models in countries other than the Netherlands and their impact on reorganizing home care services. To date, there has been no review summarizing the international state of research in this field.

## Methods

To map the existing international literature on the implementation of models based on the Buurtzorg idea in home care, we conducted a scoping review following the five stages recommended by Arksey and O'Malley (2005).

## Stage 1: Defining the research question

This scoping review aims to describe the experiences gained during the implementation of the Buurtzorg-derived model outside the Netherlands and to outline potential effects on patients and their caregivers, nurses, and the organizations or healthcare systems themselves.

## Stage 2: Identifying relevant studies

To identify a wide range of relevant studies, we adopted a systematic search strategy. We searched the electronic databases Medline, LIVIVO, CINAHL, Cochrane, JSTOR, Social Services Abstracts, SocINDEX, and PMC Europe on May 18, 2021. We used the following keywords: ("Buurtzorg" OR "Self-governing" OR "Autonomous practice") AND ("Community" OR "community care" OR "home care") AND ("nurs*" OR "registered nurses" OR "nursing team") AND "health care". The detailed search strategy and number of results are documented in the supplementary materials. As electronic databases may be incomplete and focus on research only, we performed a hand-search in relevant journals, Google Scholar, checked the bibliographies of the included studies, searched for conferences mentioning Buurtzorg, and included those materials, as far as they met our inclusion criteria.

## Stage 3: Study Selection

Publications were included if they met the inclusion and exclusion criteria described in Table 1. In the first round, titles and abstracts of the search results were reviewed independently by two researchers. In the second round, the full texts of the included studies were screened by the researchers. After a joint discussion about whether the studies corresponded to the inclusion and exclusion criteria, we decided to include or exclude them.

**Table 1:** Inclusion and exclusion criteria

Inclusion Criteria	Exclusion Criteria
- Publications describing the implementation of the Buurtzorg model and its experiences in a country other than the Netherlands - Publications describing outcomes regarding the implementation process, or outcomes on the patient level and organizational level, or impact on the healthcare system - Publications focusing on nursing or interdisciplinary teams with nurses - Home care - All study designs - Publications in English, German or French - Published from 2005 onwards	- Publications on the potential or challenges of the Buurtzorg model without reporting implementation experiences - Publications mainly focusing on pharmacists, doctors, midwifes, or physiotherapists - Focus on the clinical autonomy and self-management of patients rather than organizations/teams

## Stage 4: Charting the data

We created a charting form using Microsoft Excel that included the following details of every study that met the inclusion criteria: author(s), year of publication, scientific approach (yes or no), methods used, type of publication (e.g., articles in peer-reviewed or trade journals, conference presentations), and country of implementation (see Table 2).

**Table 2:** Characteristics of the included articles

Author (Year)	Scientific approach (yes/no)	Methods	Type of publication	Country
Brunnschweiler (2019)	No	Field report (about own home care service)	Conference Presentation	CH
Burtke (2018)	No	Field report	Trade Journal Article	DE
Cavedon et al. (2020)	Yes	Feasibility study; interviews with home care organizations working with the Buurtzorg model	Trade Journal Article	CH
Dobie et al. (2019)	Not specified	Case study report	Peer-Reviewed Journal Article	UK
Drennan, Calestani et al. (2018)	Yes	Mixed-methods case study: individual and group interviews with patients, carers, health professionals, managers; observation of nursing practice and team meetings; analysis of internal documents and anonymized patient records.	Peer-Reviewed Journal Article	UK
Drennan, Ross et al. (2018)	Yes	(Same study as described in Drennan et al. 2018)	Peer-Reviewed Journal Article	UK
Drennan et al. (2017)	Yes	(Same study as described in Drennan et al. 2018)	Report	UK
Dyckerhoff (2018)	No	Field report	Conference Presentation	Asia
Gobet and Wiederkehr (2020)	No	Field report (about own home care service)	Report	CH
Hamm and Glyn-Jones (2019)	Yes	Field report (about own home care service): open sessions with staff members, including local newsletters, professional nursing meetings, and focus groups	Peer-Reviewed Journal Article	UK
Healthcare Improvement Scotland (2019a)	Not specified	Review of local evaluation reports, case study evaluations, and stories shared by the teams	Report	UK
Healthcare Improvement Scotland (2019b)	Yes	Analysis of routinely collected data on hospital emergency (re)admissions	Report	UK
Jantunen et al. (2020)	Yes	Focus group discussions with team members, job satisfaction survey, cost development analysis with control group	Peer-Reviewed Journal Article	FIN
Klein (2018)	No	Field report (about own home care service)	Trade Journal Article	DE
Lalani et al. (2019)	Yes	Evaluation using a Researcher in Residence model. Data consisted of field notes of meeting observations, interviews with stakeholders, staff, patients/carers	Peer-Reviewed Journal Article	UK
Bell and Murray (2020)	Yes	Qualitative case study: individual interviews with staff members	Peer-Reviewed Journal Article	UK
Leask and Gilmartin (2019)	Yes	Individual interviews with patients and satisfaction questionnaire with patients	Peer-Reviewed Journal Article	UK
Lüthi (2020)	No	Field report (about own home care service)	Interview in Trade Journal	CH
Lüthi and Camenzind (2018)	No	Field report	Trade Journal Article	CH
Roloff (2019)	No	Field report	Trade Journal Article	DE
Sleziona (2019)	No	Field report (about own home care service)	Interview in Trade Journal	DE
Taillens (2020a)	No	Field report (about own home care service)	Interview in Trade Journal	CH
Taillens (2020b)	No	Field report	Trade Journal Article	CH
Wiederkehr (2016)	No	Field report	Letter to the editor in Trade Journal	CH

CH = Switzerland; DE = Germany; UK = United Kingdom; FIN = Finland

## Stage 5: Collating, summarizing, and reporting the results

The included articles were analyzed using qualitative content analysis (Kuckartz, 2016). Data analysis was performed using the content analysis software MAXQDA 2020. Two authors were involved in data analysis and the creation of categories and subcategories, as well as the coding of the data that took place in several cycles. First, the texts were carefully read to identify important segments, according to our research focus. As the coding was based on concept-driven as well as data-driven development of codes, we started out by forming main thematic categories and assigning relevant sections to these categories. Since text sections could contain several topics, multiple coding of such sections or sentences was possible. In the next step, we inductively created subcategories and subsequently ordered and systematized them. Similarities and differences in text segments were then easily identified. Based on this, we performed the analysis. The resulting topics and their subtopics are discussed in the next section.

## Results

### Selection process and characteristics of included sources

**Figure 1:**
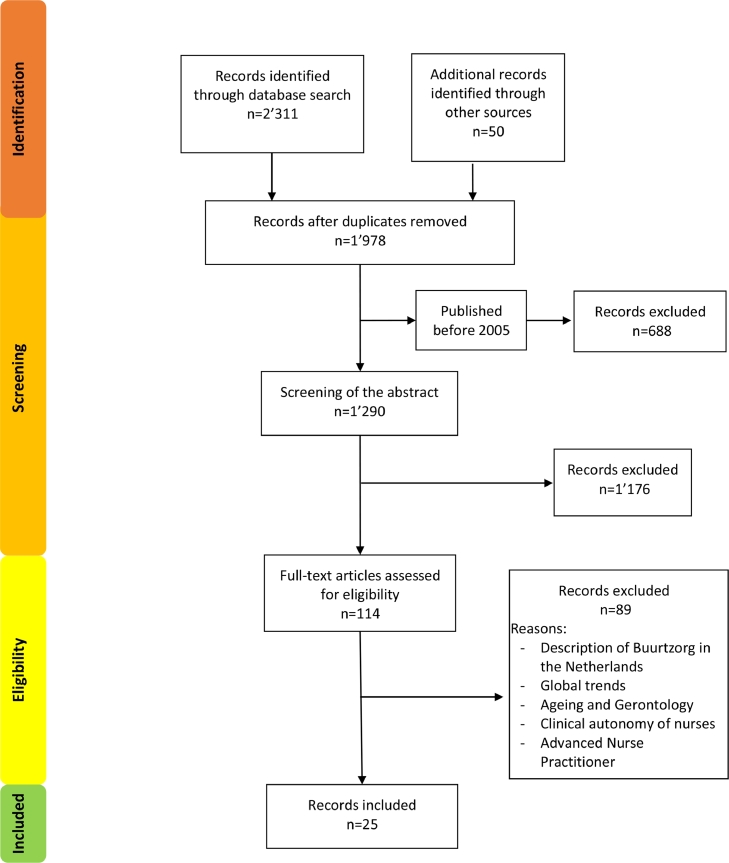

The study selection process is illustrated in Figure 1. After screening 1290 abstracts for eligibility, 114 full texts were selected for a thorough assessment. Overall, 25 records were included in the scoping review.

The characteristics of the included publications are detailed in Table 2. The experiences and potential effects reported by the included publications could be allocated to the topics and subtopics, as described in the following section.

## Experiences and potential effects

### Understanding of care and nursing practice

Nurses working with Buurtzorg-derived models adopted a holistic approach to care. They performed various tasks and activities that are associated with public and community health nursing and implemented a multidisciplinary approach that spanned health and social care (Bell and Murray, 2020; Drennan et al., 2017; Drennan, Calestani et al., 2018; Dyckerhoff, 2018; Healthcare Improvement Scotland, 2019a, 2019b; Lalani et al., 2019; Leask and Gilmartin, 2019).

Most authors emphasized that nurses were not only expected to complete nursing tasks in a competent and pleasant manner but also specifically focus on patients' needs by enabling independence, self-care, building relationships, and including the patients' social networks (Drennan et al., 2017; Hamm and Glyn-Jones, 2019; Healthcare Improvement Scotland, 2019a; Lalani et al., 2019; Leask and Gilmartin, 2019; Sleziona, 2019). Therefore, nurses need to spend extended periods of time with the patients and create a sense of familiarity (Hamm and Glyn-Jones, 2019; Lalani et al., 2019; Sleziona, 2019) as well as integrate health and social care. This requires coworking and a new distribution of the tasks among professionals and the patient's social support system (Dobie et al., 2019; Drennan et al., 2017; Healthcare Improvement Scotland, 2019a; Klein, 2018; Leask and Gilmartin, 2019; Roloff, 2019; Taillens, 2020a).

In addition, their work was characterized by compassion (Leask and Gilmartin, 2019) and a proactive approach (Drennan et al., 2017), by using a wide range of opportunities to address the needs of patients and caregivers (Drennan et al., 2017). Some articles described how staff was effective in linking patients to community resources and how unpaid carers were involved in decisions (Healthcare Improvement Scotland, 2019a; Lalani et al., 2019; Leask and Gilmartin, 2019) and health care activities (Roloff, 2019). This promoted an open dialog between staff and families and facilitated patients' access to external support structures (Healthcare Improvement Scotland, 2019a; Leask and Gilmartin, 2019).

Having full control over the frequency and timing of visits allowed nurses to work with a more person-centered approach (Bell and Murray, 2020). Overall, care seemed to be less fragmented and more flexible. It allowed support to persons with multiple complex needs (Healthcare Improvement Scotland, 2019a).

### Relationship and nurse-patient communication

Various studies reported an improved continuity of care (Bell and Murray, 2020; Brunnschweiler, 2019; Drennan et al., 2017; Drennan, Calestani et al., 2018; Drennan, Ross et al., 2018; Lalani et al., 2019) that had an impact on how patients' needs were addressed and consequently impacted patients' clinical care and health (Drennan et al., 2017; Drennan, Calestani et al., 2018; Drennan, Ross et al., 2018). For example, a study reported that continuity of care allowed nurses to account for changes in needs more quickly and therefore, provided a high-quality service (Bell and Murray, 2020).

Several studies have reported changes in communication between nurses and patients. For example, patients were able to directly contact the nurses via telephone, without reception or phone systems (Drennan, Calestani et al., 2018; Drennan, Ross et al., 2018; Lalani et al., 2019). Consequently, the teams were able to address complaints directly (Cavedon et al., 2020). In addition, Drennan et al. (2017) found that increased telephone consultations and follow-ups were reported in the Buurtzorg-model nursing teams than in the traditional nursing services.

Continuous, personal, and caring relationships between patients, their families, and nurses could be developed or strengthened (Bell and Murray, 2020; Cavedon et al., 2020; Drennan et al., 2017; Drennan, Calestani et al., 2018; Roloff, 2019). It was noted that patients knew the team members and vice-versa (Brunnschweiler, 2019; Drennan et al., 2017), relationships could be built even with patients/carers who were disengaging with health professionals (Drennan et al., 2017), and that a sense of familiarity and trust was created (Lalani et al., 2019; Leask and Gilmartin, 2019; Roloff, 2019). These relationships enabled patients and their caregivers to make informed decisions about their own care (Healthcare Improvement Scotland, 2019a) and contributed to the patients' re-ablement process and strengthened their self-efficacy (Leask and Gilmartin, 2019). However, Drennan et al. (2017) highlighted that some patients might find close personal relationships intrusive. Therefore, it is important to respect expectations and preferences.

### Networking, collaboration, and integration into community

One of the central characteristics of Buurtzorg is anchoring in the community and de-centralized working. Therefore, the geographical radius of the teams was sometimes limited to walking distance from the office or team members' homes to the patients (Burtke, 2018; Drennan, Ross et al., 2018).

Various articles mentioned nursing teams' efforts to network and build close connections with local (para)medical and social care professionals as well as the patients' social support systems such as family caregivers, neighbors, or community groups (Brunnschweiler, 2019; Burtke, 2018; Drennan, Ross et al., 2018; Healthcare Improvement Scotland, 2019a; Klein, 2018; Leask and Gilmartin, 2019; Roloff, 2019; Sleziona, 2019; Taillens, 2020a). Studies reported that teams who worked with Buurtzorg-derived models received positive feedback from other health professionals. For example, for being enthusiastic and caring for patients; moreover, effective professional collaborations and trusting relationships could be developed to support care coordination (Drennan et al., 2017; Healthcare Improvement Scotland, 2019a; Lalani et al., 2019; Leask and Gilmartin, 2019).

However, it was challenging to make decisions beyond their own profession as cross-professional working and separate budgets often did not allow the authorization of resource use from other professions (Healthcare Improvement Scotland, 2019a). In addition, Drennan et al. (2017) reported some communication issues regarding nurses differentiating between issues that needed the urgent attention of a general practitioner and those that were less urgent.

The co-location of teams, i.e., physically locating all members of the team in one place, was beneficial in establishing common work processes, sharing roles and responsibilities, and improving IT access, communication, and information sharing (Bell and Murray, 2020; Healthcare Improvement Scotland, 2019a). Co-location within a general practice or shared care records could potentially promote effective collaborations (Bell and Murray, 2020; Drennan et al., 2017). According to several authors, weekly or monthly team meetings were complemented by newly established multidisciplinary meetings (Dobie et al., 2019; Lalani et al., 2019). Meetings were carried out with a solution-oriented focus, but Drennan et al. (2017) reported that the first meetings were lengthy, and the teams were dealing with administrative matters rather than discussing patient care. Nevertheless, these locally based teams and multidisciplinary meetings have improved care coordination (Cavedon et al., 2020; Dobie et al., 2019; Healthcare Improvement Scotland, 2019a).

### Self-managed teams

**Roles within the team.** Since the teams were self-managed, team members had specified responsibilities, such as staffing rota, chairing, and writing minutes at the team meetings. However, they also took on less-specified roles (e.g., being the expert on IT aspects, providing positive feedback, and appreciating other members). These responsibilities are usually rotated to ensure that each team member is exposed to non-clinical activities (Brunnschweiler, 2019; Drennan et al., 2017; Lalani et al., 2019). The devolution of responsibility to the nursing team of organizing rotas, shifts, weekend cover, and annual leave mainly worked well, despite initial reservations from the organizations (Hamm and Glyn-Jones, 2019).

Autonomy in managing rotas and shifts was seen as a key feature of the Buurtzorg model (Lalani et al., 2019) and allowed the teams to be agile in delivery of care and to escalate or de-escalate the frequency and duration of support. This might also contribute to reducing the pressure of time faced by community health nurses (Lüthi, 2020).

The team members also undertook activities that would normally be assigned to managers, such as the recruitment of new staff. Being involved in this type of activity was described in positive terms (Drennan et al., 2017), but nurses also wished for some sort of lead to "drive forward" (Bell and Murray, 2020). In addition, some organizations required a manager's signature for certain administrative duties, budgets, or ordering supplies, which was perceived as contradictory and frustrating by the self-managed nursing team (Drennan et al., 2017; Hamm and Glyn-Jones, 2019).

Hamm and Glyn-Jones (2019) highlighted that great care needs to be taken not to destabilize the workforce during the reorganization process. This major change in roles and responsibilities is a significant structural change that can bring about a deep sense of personal loss.

**Shared decision-making and assuming responsibility.** Many articles have pointed out the team's collective responsibility for the outcomes of their work. Teams were expected to develop their own solutions within the national and organizational frameworks. Various authors highlighted that decision-making was based on consensus, and changes were adopted when team members agreed (Brunnschweiler, 2019; Dobie et al., 2019; Drennan et al., 2017; Drennan, Calestani et al., 2018; Hamm and Glyn-Jones, 2019). However, some authors emphasized that the engrained hierarchical tradition within nursing required staff members to "unlearn" dependent behavior toward managers and to "learn" how to make and accept decisions as a team and shift an established mindset to perceiving junior nurses as equal partners (Brunnschweiler, 2019; Cavedon et al., 2020; Dobie et al., 2019; Drennan, Calestani et al., 2018; Jantunen et al., 2020; Lalani et al., 2019; Lüthi and Camenzind, 2018). Team leaders could positively influence team members toward self-organization if they were committed and fully advocating for the same (Jantunen et al., 2020). However, teams needed support and continuous reflection on their practice to transform their way of working from a hierarchically managed approach to authentic self-management (Gobet and Wiederkehr, 2020; Hamm and Glyn-Jones, 2019). This support was provided by coaches but also through training courses (e.g., training in communication skills, IT, and administration or training for housekeepers to become healthcare assistants; Bell and Murray, 2020; Cavedon et al., 2020; Drennan et al., 2017; Gobet and Wiederkehr, 2020; Lalani et al., 2019; Lüthi and Camenzind, 2018; Sleziona, 2019). However, Lalani et al. (2019) reported that teams possibly created some extra work by not accessing the relevant training provided by the organization.

**Team composition and skills development.** As previously described, adopting a self-managed work style is one of the main challenges of the implementation of Buurtzorg-derived models. Having the right mix of experience in the nursing team was seen as important in achieving quality (Drennan et al., 2017). However, while Drennan et al. (2017) reported the need to have experienced staff in the team, varying qualifications and experience could also hinder the development of effective working relationships (Lalani et al., 2019). Nurses who had no previous experience in the home care setting needed to learn about practicing nursing in the community and in people's homes (Drennan et al., 2017).

Moreover, nurses working in self-managed teams needed to have the ability to work in a team, accept criticism, have strong communication skills, creativity, the joy of decision making, and a willingness to renounce and adapt (Brunnschweiler, 2019; Lüthi and Camenzind, 2018; Sleziona, 2019). An important skill required to support personal growth is dialog that enables participants to freely exchange their interpretations and viewpoints, giving space for new kinds of knowledge to emerge (Jantunen et al., 2020). Additionally, Lalani et al. (2019) suggested that nurses should have good IT skills, as the Buurtzorg model promotes mobile working and IT proficiency. However, these skills are not pre-conditions and can be obtained and practiced through work or organizational training (Sleziona, 2019). Nevertheless, self-managing teams with a flat, non-hierarchical structure may not be acceptable to all nurses (Bell and Murray, 2020; Cavedon et al., 2020; Drennan et al., 2017). Persons with pronounced dominant behavior and those who avoid a higher pressure of responsibility may not be suitable for a Buurtzorg-team (Cavedon et al., 2020).

The main responsibility for the patient lies with the primary nurse, who was often supported by an associated nurse in the team (Drennan et al., 2017). In contrast to the Buurtzorg Netherland teams, some teams consisted of nurses with various grades of higher education (diploma, bachelor), assistants with vocational education certification, or home health aides with short courses (Brunnschweiler, 2019; Dobie et al., 2019; Klein, 2018). Some Buurtzorg-teams included not only nurses but also housekeepers or support workers (Bell and Murray, 2020; Lüthi and Camenzind, 2018). Tasks that do not necessarily require a registered nurse can be increasingly performed by persons from patients' social environments (Burtke, 2018).

The non-hierarchical nature of the team allowed team members to develop new skills or build experience in activities usually reserved for staff of certain grades (e.g., recruitment process; Drennan et al., 2017). Developing a workforce to have confidence in itself was also reflected in attitudes toward career development. Hamm and Glyn-Jones (2019) reported a discernible new attitude by nurses seeking promotion, which was noticeably proactive. In response, a new career framework has been developed that is linked to clinical skills and experience (Hamm and Glyn-Jones, 2019). Conversely, Drennan, Calestani et al. (2018) reported issues regarding the nature of "flat" structures with salaries fixed on previous employments and no clarity on career and financial progression.

### Support services (back office)

Similar to Buurtzorg Netherlands, most teams were supported by coaches and back-office services (e.g., ICT, administration, human resources, procurement) in their activities. These support structures were either already set into place before the transition to the Buurtzorg model (Brunnschweiler, 2019) or were established later on (Drennan et al., 2017). Drennan et al. (2017) recommended the establishment of back-office services on day one.

**IT Support.** While Buurtzorg Netherlands has an established IT infrastructure, some models in other countries still worked on the development of such systems (Brunnschweiler, 2019; Drennan, Calestani et al., 2018; Gobet and Wiederkehr, 2020; Healthcare Improvement Scotland, 2019a). Others provided their teams with tablet computers, a cloud system, and an intranet that allowed the team members to connect with other team members (Klein, 2018), worked with secured WhatsApp groups to establish contact between the coaches and the teams (Taillens, 2020b), or used their work phones and email to write care notes (Drennan et al., 2017). Missing IT services, missing access for support workers, or IT that was not applicable to mobile working in the community were described as irksome (Bell and Murray, 2020; Drennan, Calestani et al., 2018). Some of the team members would have liked to have the opportunity to be part of activities to improve access and use of IT to aid nursing practice as well as administrative needs (Drennan et al., 2017).

**Coaches.** The role of coaches was to facilitate the teams to be self-managed and were as important as the back office (Drennan, Calestani et al., 2018). Coaches offered assistance in communication, collaboration, organization, distribution of roles and tasks (Bell and Murray, 2020; Drennan, Calestani et al., 2018; Lalani et al., 2019; Leask and Gilmartin, 2019; Sleziona, 2019) and acted as "buffers" between the greater organization and the Buurtzorg-teams (Drennan, Calestani et al., 2018; Hamm and Glyn-Jones, 2019). Especially at the beginning of the transformation, coaches needed to help in terms of problem-solving and to support cultural change (Drennan et al., 2017; Lalani et al., 2019).

In the course of reorganizing from a traditional to a Buurtzorg organization, some of the executive personnel could be transferred to the back office services or as coaches (Cavedon et al., 2020; Hamm and Glyn-Jones, 2019; Lüthi and Camenzind, 2018). In this case, training for coaches might be considered to facilitate role change (e.g., abandoning the reflex to quickly offer a solution when faced with challenges) (Cavedon et al., 2020).

### Staff retention, onboarding

In some Buurtzorg-derived models, team members were responsible for recruiting new staff. In addition to hard criteria (such as degrees, qualifications, salary expectations, and experiences), sympathy was important in the recruitment process (Sleziona, 2019). Hamm and Glyn-Jones (2019) were successful in using a strength-based and values-focused approach for recruitment. Selection was centered on staff who could think outside the box, were innovators, and not just leaders, but also followers. Lalani et al. (2019) reported a recruitment process through non-standardized approaches, including an assessment day conducted by individuals, that are associated with and external to the Buurtzorg-derived model.

While some articles reported that the recruitment of new staff was successful (Brunnschweiler, 2019; Drennan et al., 2017; Drennan, Calestani et al., 2018), others had difficulties (Bell and Murray, 2020). Vacancy rates could be reduced, (Drennan et al., 2017) and one organization reported receiving more applications (Brunnschweiler, 2019). In contrast, institutions experienced that some potential applicants declined the work after the job interview or left the post early after hiring (Bell and Murray, 2020; Drennan, Calestani et al., 2018). However, team dynamics hindered the development of effective working relationships among nurses (Lalani et al., 2019). While some authors reported a stable workforce (Brunnschweiler, 2019; Gobet and Wiederkehr, 2020; Lüthi and Camenzind, 2018), high turnover of staff was evident where team members were inexperienced and uncomfortable working in a self-managing way (Bell and Murray, 2020).

### Staff and patients' satisfaction

Various studies have reported increased staff satisfaction (Jantunen et al., 2020; Klein, 2018; Roloff, 2019; Sleziona, 2019; Wiederkehr, 2016) due to the staff's possibility to upskill (Bell and Murray, 2020) to participate in decisions (Klein, 2018; Lalani et al., 2019), the teamwork including a collective problem-solving approach (Drennan et al., 2017; Drennan, Calestani et al., 2018), the (holistic) care delivered to the patients (Drennan et al., 2017; Drennan, Ross et al., 2018; Lalani et al., 2019), increased self-confidence, (Cavedon et al., 2020) and the positive feedback received from patients, caregivers, and other health professionals (Drennan et al., 2017; Drennan, Ross et al., 2018). Other studies have reported difficulties faced by the staff, owing to low caseloads that had a negative impact on their self-worth, high caseloads that overburdened them, a greater administrative burden, and a lack of work-life-balance due to constant availability and frequent team meetings (Bell and Murray, 2020; Lalani et al., 2019).

Most studies included in the review reported that patients were satisfied with the care delivered through the Buurtzorg-teams (Brunnschweiler, 2019; Drennan et al., 2017; Drennan, Calestani et al., 2018; Leask and Gilmartin, 2019; Lüthi and Camenzind, 2018; Sleziona, 2019; Wiederkehr, 2016). Drennan et al. (2017) reported that there were no complaints from patients or that the complaints could be dealt with satisfactorily. Reasons for patient satisfaction were continuity of care (Drennan, Calestani et al., 2018; Lalani et al., 2019), continuous and intensive relationships with the nurses (Drennan et al., 2017), team members' agility in care delivery (Leask and Gilmartin, 2019), sufficient time with patients (Klein, 2018), responsible staff members (Leask and Gilmartin, 2019), staff's proactive support and problem-solving strategies, willingness to listen (Drennan, Calestani et al., 2018; Drennan, Ross et al., 2018), information about their care delivery, (Leask and Gilmartin, 2019) and improved accessibility of team members (Drennan, Calestani et al., 2018; Lalani et al., 2019; Leask and Gilmartin, 2019).

### Effects on patients' outcomes

The included articles reported benefits to patients' well-being (Dobie et al., 2019; Drennan, Ross et al., 2018; Leask and Gilmartin, 2019), health (Cavedon et al., 2020; Dobie et al., 2019; Drennan et al., 2017; Drennan, Calestani et al., 2018; Drennan, Ross et al., 2018), self-efficacy (Leask and Gilmartin, 2019), self-management (Healthcare Improvement Scotland, 2019a), confidence (Drennan et al., 2017), and positive changes in lifestyle behavior (Leask and Gilmartin, 2019).

### Costs

Some articles commented on their limited capability to assess cost efficiency (Drennan et al., 2017; Healthcare Improvement Scotland, 2019b). However, there seemed to be an impact on adjacent nursing organizations with a reduction in workload (Drennan et al., 2017). Jantunen et al. (2020) could not identify any positive changes in costs after one year. However, they argued that changes in costs require more time and are difficult to measure due to the dynamics of home care, for example, through changes in the number of patients.

More detailed observations reported that the Buurtzorg model achieved an efficient system of recording by streamlining record keeping. For example, by making it more meaningful to practice or making brief notes only in the case of changes in the care plan or medication (Drennan et al., 2017; Drennan, Ross et al., 2018). However, patients noted the excessive paperwork that was done by the nurses during the visits (Leask and Gilmartin, 2019).

Some studies assumed some improvements related to costs, such as savings in administration costs (Cavedon et al., 2020) and costs associated with hospital admissions (Healthcare Improvement Scotland, 2019b), reduced rates of emergency hospital admissions (Healthcare Improvement Scotland, 2019b; Lalani et al., 2019), reduced hours of care (Sleziona, 2019), decreasing duplications through improved care coordination (Lalani et al., 2019), increased productivity, (Lüthi and Camenzind, 2018) and reduced time needed for the drive to the patient's home (Burtke, 2018; Roloff, 2019). However, these results were not conclusive and varied between the study sites.

### Discussion

This scoping review reflects the discourse on the Buurtzorg model after the advent of the international home and community care arena. A wide range of scientific and non-scientific publications have reported on the experiences of home care services implementing a Buurtzorg-derived model outside the Netherlands. The implementation approaches focused mainly on structural and staffing aspects (self-managed teams, support services, collaboration with other providers) and resulted in changes toward person-centered care and satisfaction among nurses and patients. The main challenges were related to the self-managed working culture, organizational framework, and national health care policies that hinder the implementation process.

### Staffing and team dynamics

One of the outstanding features of Buurtzorg is the self-managed teams aiming to provide continuity of care, resulting in job enrichment within locally embedded nursing care experiences. Historically, healthcare institutions and nursing services in particular were (and still are) organized in hierarchical structures. Therefore, developing the competencies for self-organization and professional accountability for the well-being of teams as well as patients and families requires not only new skills but also a new mindset (van Eenoo et al., 2018).

Additionally, depending on reimbursement schemes based on local health policies, home care teams may consist of different professional groups (e.g., social or support workers, facility managers, housekeepers) and various grades among registered nurses and associates or auxiliary staff. This team structure differs from those of Buurtzorg Netherlands and leads to a division of labor within an organization that needs constant negotiation about sharing and delegating tasks. Based on these findings, managing various skills and grades within a team requires developing a new mindset during the Buurtzorg implementation process and constant awareness of coaching and learning opportunities within an organization (World Health Organization, 2014). This endeavor can already be considered in the recruitment of new team members — for example, searching for applicants who are self-reliant, have person-centered communication and management skills, and are open to changing the traditional mindset.

Furthermore, nurses need to be skilled in community health nursing, particularly regarding health promotion, disease prevention and management (World Health Organization, 2017). The studies included in this review reported experiences from the first years of the implementation of the Buurtzorg-model and, thus, rarely mentioned the public health aspects of community health nursing that are, however, central to the Buurtzorg model in the Netherlands. The shift from care provision to health promotion and disease prevention in home care services is a profound change that can take several years to complete and needs policy actions as well. Organizations aiming to implement the Buurtzorg-model should at least make sure that their nurses have basic competencies and experiences in community health nursing.

However, this proves challenging due to the workforce shortage in nursing; this shortage and the consequences have been highlighted in the COVID-19 pandemic. Although Buurtzorg stakeholders promise attractive, innovative working conditions, our review shows that recruiting and onboarding can be challenging for individual teams and the entire home care service. Additionally, blurred boundaries between work and leisure time challenge staff retention in home care services applying the Buurtzorg-model (Bell and Murray, 2020). However, team members' satisfaction was high after the implementation of the Buurtzorg principles. Ideally, when Buurtzorg-derived systems are sustainably established and new structures settled, Buurtzorg or other innovative concepts following a person-centered approach might contribute to staff shortages by adding to the diversity of home care services, motivating nurses to work in the community setting and stay in the job (de Silva, 2014).

### Challenging IT implementation

In addition to the skills and mindset of the workforce, Buurtzorg-derived models require an established IT infrastructure to process staffing, administrative, and financial matters efficiently. Our review showed that the models reported difficulties due to inappropriate or not yet fully developed and established IT systems. The challenges of implementing technology in home care settings are well known but have not been fully overcome in heterogeneous, often small-scale home care services (Renyi et al., 2020). According to the framework of non-adoption, abandonment, and challenges to scale-up, spread, and sustain technology-supported change efforts in health and social care, the complexity of projects and systems determines the success of technology (Greenhalgh and Abimbola, 2019). Namely, the more complexity there is in the system, the less likely the technology is to achieve sustained adoption. Since home care is a complex setting and Buurtzorg-derived models are complex interventions, convincing leadership strategies are needed to handle this complexity. For example, by allowing for extra time for implementation and by involving users – mainly nursing teams – into technology development. This is essential for facilitating efficient implementation processes in home care services.

### Increasing quality of care to the next level

The home care sector is increasingly facing care needs of people with multimorbidity (Rijken et al., 2016); this situation leads us to the question of how to ensure quality of care for those with complex care needs. In primary health care, providing a comprehensive and intersectoral range of services is critical in the promotion of health and well-being (World Health Organization, 2018). Therefore, this framework is relevant for achieving the intended effects for Buurtzorg-derived models.

The results from our review illustrate that flexibility and continuity of care foster a person-centered approach for patients with complex care needs. In addition, home care services with a broader disciplinary approach, such as an approach that incorporates social and health care, might improve the management of complex care needs (Cameron et al., 2014). However, the quality of care and outcomes of care are rarely mentioned or assessed. Even though person-centeredness might result in better care delivery and outcomes (de Silva, 2014), explicit quality standards are necessary. These standards might differ between countries but are essential when caring for patients across all ill-health conditions. One way to ensure the quality of care for patients with multiple illnesses, such as Long-COVID (Razai et al., 2021), is the integration of advanced practice nurses into home care teams (Trilla et al., 2018). Currently, and internationally, there is a lack of information on this nursing grade level in Buurtzorg-teams. Yet, to cover the entire spectrum of health care needs at home, including a wide range of pharmaceutical and interventional therapies, coordination of multiple providers, and even "Hospital@Home", where hospital treatments are shifted to the private home (Caplan et al., 2012), the implementation of advanced practice nurses roles in the Buurtzorg staffing model is needed. This integration requires rather generalized expertise in advanced nursing practice rather than the highly specialized expertise common in hospital care (Jenkins et al., 2021).

### Strengths and Limitations

To the best of our knowledge, this review is the first to address the implementation of the Buurtzorg model outside the Netherlands. Our extensive database and hand search, plus the inclusion of all publication types in English, German, and French, resulted in a sound database. However, we might have missed some publications, especially trade journal articles that were not indexed in online databases. In addition, if publications did not explicitly state a connection to the Buurtzorg model, we might have missed them in the selection process. At least two people were involved in data analysis and the coding process to ensure the reliability of the categories. However, we included all publications on the topic without critical appraisal of the studies. Therefore, the quality and significance of the evidence varied.

### Multilevel implications and conclusions

If home care services plan to develop requirements and business changes toward Buurtzorg principles, they need to be aware of multiple level implications: the implementation process of self-managed teams, the preparation of flawless IT support, and a culture change mindset (Buurtzorg, 2021a; Drennan, Calestani et al., 2018).

Local small teams need to be supported by the central office to recruit and select team members as well as to scale-up the necessary skill set (communication, innovation, accountability). Home care providers and healthcare policymakers must create an innovative working environment and business conditions that promote self-management and continuity of care as a standard service (Jantunen et al., 2020).

The adaptation and implementation of Buurtzorg principles outside the Netherlands is an ongoing and dynamic process. This process is currently the focus of a research project that aims to present a methodology to overcome blocking points in transferring socially innovative service models from one area to another (The Health and Europe Centre, 2021). Implementation processes should be accompanied by an evaluation and research approach. For example, implementation research or action research methods can be applied to address the middle- and long-term changes that allow examination of the effects of sustainability on staffing and person-centered care outcomes (de Silva, 2014). In particular, reliable findings on cost-effectiveness and patient and family carer-relevant outcomes from outside the Netherlands are lacking. In addition, following the person-centered principles of Buurtzorg, future research should adopt a participatory approach by including patients and family caregivers as co-researchers and consequently improve the relevance of research (Morrow, 2012).

Further implications for research are derived from the COVID-19 crisis, the resulting increasing staff need, and the overburdening of home care teams and family caregivers (Kricheldorff, 2020). Until now, there has been little data and insights from Buurtzorg home care services on how COVID-19 influenced staff retention and their efforts to mobilize community resources to support patients.

In many Western countries, health care policies are striving to shift inpatient to outpatient care — even more so since the COVID-19 pandemic began. The crisis has highlighted the need for a much smoother transition of ill or injured patients to home health care. Consequently, sustainable home care staff, IT structures, and finances need to be established within the health care system and across intersectoral systems following recommendations from the WHO Alma Ata Charta on primary health care back in 1978 (World Health Organization, 1978), and repeatedly urged calls from the World Health Organization (Tello et al., 2018).

Finally, health care educationalists need to consider the principles of Buurtzorg on all education levels to prepare the future health care workforce for more self-managed working environments, engaged patients, and family members. They all have to be competent with innovative primary health care service models, and in particular, deal with IT systems and digital health.

In conclusion, Buurtzorg is a complex intervention that requires a long-term vision for practice development. Consequently, it can be concluded that there is no quick fix for Buurtzorg-related changes in home care. Rather, similar to individualized care, an individualized approach and solid conceptual preparation for implementation in local organizations are needed.

## Funding sources

This research did not receive any specific grant from funding agencies in the public, commercial or not-for-profit sectors.

## Author Contributions

AH conceptualized the project with input from AS and IB. AH and AS were involved in the search, data extraction and data analysis. All authors were involved in data interpretation and in editing and approving the manuscript.

## Declaration of Competing Interest

The authors declare to have no conflicts of interest.

## Appendix Supplementary materials

Image, application 1
