# Supplementary material for: Implementing Buurtzorg-derived models in the home care setting: a Scoping Review
Source: Int J Nurs Stud Adv. 2022 Jan 12;4:100061. doi: 10.1016/j.ijnsa.2022.100061 (PMC11080323; doi:10.1016/j.ijnsa.2022.100061)
Supplement: Supplementary file 1 [file mmc1.docx]

| **Database** | **Keywords combined with AND/OR** | **Limitations** | **Results** |
| --- | --- | --- | --- |
| Medline (Pubmed) | Buurtzorg |  | 21 |
|  | (Buurtzorg OR (Self governing) OR (Autonomous practice)) AND (Community OR (community care) OR (home care)) AND (nurs* OR (registered nurses) OR (nursing team)) AND (health care) |  | 504 |
| LIVIVO | Buurtzorg | without Medline | 30 |
|  | (Buurtzorg OR (Self governing) OR (Autonomous practice)) AND (Community OR (community care) OR (home care)) AND (nurs* OR (registered nurses) OR (nursing team)) AND (health care) | without Medline, only article | 102 |
| CINAHL | Buurtzorg |  | 24 |
|  | (Buurtzorg OR (Self governing) OR (Autonomous practice)) AND (Community OR (community care) OR (home care)) AND (nurs* OR (registered nurses) OR (nursing team)) AND (health care) |  | 88 |
| Cochrane | Buurtzorg | all Text or Title, Abstract, Text | 0 |
|  | (Buurtzorg OR (Self governing) OR (Autonomous practice)) AND (Community OR (community care) OR (home care)) AND (nurs* OR (registered nurses) OR (nursing team)) AND (health care) | all Text, Reviews | 483 |
|  | (Buurtzorg OR (Self governing) OR (Autonomous practice)) AND (Community OR (community care) OR (home care)) AND (nurs* OR (registered nurses) OR (nursing team)) AND (health care) | all Text, Trials | 58 |
|  | (Buurtzorg OR (Self governing) OR (Autonomous practice)) AND (Community OR (community care) OR (home care)) AND (nurs* OR (registered nurses) OR (nursing team)) AND (health care) | Title, Abstract, Text, Reviews | 2 |
|  | (Buurtzorg OR (Self governing) OR (Autonomous practice)) AND (Community OR (community care) OR (home care)) AND (nurs* OR (registered nurses) OR (nursing team)) AND (health care) | Title, Abstract, Text, Trials | 53 |
| JSTOR | ((Buurtzorg OR (Self governing) OR (Autonomous practice)) AND (Community OR (community care) OR (home care)) AND (nursing team) AND (health care)) |  | 878 |
|  | Buurtzorg |  | 11 |
| Social Services Abstracts | Buurtzorg |  | 1 |
| SocioIndex | Buurtzorg |  | 0 |
| PMC Europe | Buurtzorg |  | 56 |
